# Ototoxicity of Non-aminoglycoside Antibiotics

**DOI:** 10.3389/fneur.2021.652674

**Published:** 2021-03-09

**Authors:** Leonard P. Rybak, Vickram Ramkumar, Debashree Mukherjea

**Affiliations:** ^1^Department of Otolaryngology, Southern Illinois University School of Medicine, Springfield, IL, United States; ^2^Department of Pharmacology, Southern Illinois University School of Medicine, Springfield, IL, United States

**Keywords:** ototoxicity, vancomycin, erythromycin, capreomycin, chloramphenicol, azithromycin, clarithromycin, macrolides

## Abstract

It is well-known that aminoglycoside antibiotics can cause significant hearing loss and vestibular deficits that have been described in animal studies and in clinical reports. The purpose of this review is to summarize relevant preclinical and clinical publications that discuss the ototoxicity of non-aminoglycoside antibiotics. The major classes of antibiotics other than aminoglycosides that have been associated with hearing loss in animal studies and in patients are discussed in this report. These antibiotics include: capreomycin, a polypeptide antibiotic that has been used to treat patients with drug-resistant tuberculosis, particularly in developing nations; the macrolides, including erythromycin, azithromycin and clarithromycin; and vancomycin. These antibiotics have been associated with ototoxicity, particularly in neonates. It is critical to be aware of the ototoxic potential of these antibiotics since so much attention has been given to the ototoxicity of aminoglycoside antibiotics in the literature.

## Introduction

Aminoglycoside antibiotics are well-known to cause ototoxicity, particularly hearing loss. Other antibiotics can also cause significant ototoxicity but the effects of these drugs on hearing and balance have not received the same widespread attention as have the aminoglycosides. This paper reviews the ototoxicity of other antibiotics to emphasize that these drugs also can cause hearing loss and balance disturbances. The antibiotics included are capreomycin, erythromycin, azithromycin, clarithromycin and vancomycin. This review was carried out by searching PUBMED using the following search terms: capreomycin ototoxicity, erythromycin ototoxicity, azithromycin ototoxicity, clarithromycin ototoxicity, macrolide ototoxicity, vancomycin ototoxicity and chloramphenicol ototoxicity.

## Capreomycin Ototoxicity

### Clinical Studies

Capreomycin is a cyclic polypeptide antibiotic that was isolated from *Streptomyces capreolus* (1). It belongs to an important class of antibiotics called the tuberactinomycins. It inhibits bacterial protein synthesis and is active against multidrug-resistant tuberculosis (MDR-TB) (2). This alarming phenomenon emerged as a clinical entity in the early 1990s (3). This worldwide problem is increasing in incidence (4). Second-line injectable agents including aminoglycosides and capreomycin in combination with fluoroquinolones, form the backbone for the treatment of MDR-TB (5). Treatment of multidrug-resistant tuberculosis (MDR-TB) is challenging and requires extensive and prolonged treatment with multidrug combinations for up to 2 years (6). Even more ominous is the finding that between 5 and 10% of MDR-TB cases are thought to be due to extensively drug resistant tuberculosis (XDR-TB) i.e., infections resistant to rifampicin, isoniazid, any fluoroquinolone and one of the second line injectable agents (7).

Ototoxicity associated with capreomycin in humans has been demonstrated in numerous publications. In a series of 294 patients two patients developed sensorineural hearing loss (8). In another series of patients who received capreomycin in Japan developed high frequency sensorineural hearing loss in 3 of 93 patients with initial treatment and in 6 of 64 cases undergoing retreatment (9). Sixty-four patients with MDR-TB were treated with either aminoglycosides alone or aminoglycosides followed by capreomycin were monitored with audiograms. One of the four patients in the latter group developed high frequency sensorineural hearing loss (4). Another study of patients in the U.K. with MDR-TB showed that a small number of patients treated with injectable capreomycin alone had no hearing loss (0/11) whereas two patients (2/5) treated with amikacin plus capreomycin demonstrated hearing loss. These findings suggest that combinations of capreomycin with aminoglycosides increase the likelihood of ototoxicity (10).

An investigation of 612 patients with confirmed or presumed MDR-TB treated with aminoglycosides or capreomycin was carried out in two hospitals in Ethiopia. Hearing loss was determined by clinician diagnosis or patient report but was not confirmed by audiograms. Symptomatic hearing loss was higher with amikacin/kanamycin (14%) compared with capreomycin (4.2%) (11). Increased hearing loss in patients treated with both capreomycin and amikacin was also reported by (6). They reviewed the records of 100 consecutive patients treated for MDR-TB at four MDR-TB centers in the U.K. They found that 2/30 (7%) patients treated with capreomycin only developed hearing loss, whereas 3/7 (43%) treated with capreomycin followed by amikacin lost hearing (6). These findings were confirmed with serial audiograms.

A prospective and retrospective study of 60 patients treated for MDR-TB with aminoglycosides or capreomycin at two hospitals in India from 2011 to 2016 found that 20% of capreomycin treated patients demonstrated permanent high frequency sensorineural hearing loss. Ototoxicity was associated with a large cumulative dose and older age (12). Records of 115 South-African patients with XDR-TB were retrospectively reviewed. This group included both HIV+ and HIV- patients. Ten patients treated with capreomycin suffered hearing loss. Surprisingly, only 2/48 (4%) HIV+ patients had hearing loss, whereas 8/67 (12%) HIV- patients experienced hearing loss even though HIV+ patients seem to be more susceptible to other adverse effects of capreomycin (7).

The Uppsala Monitoring Center (UMC) is the World Health Organization Collaborating Center for international drug monitoring. It maintains a repository of data on adverse drug reports from around the world known as VigiBase®. This database contains individual case safety reports (ICSRs). The UMC conducted a study of patients treated with aminoglycosides or capreomycin for the treatment of drug-resistant TB who developed ototoxicity related to treatment. They analyzed all ICSRs in VigiBase® from 56 countries, mostly from Asia and Europe between 1968 and June 2014 where these drugs were administered. Authors utilized logistic regression analysis to determine the association between the administration of parenteral antibiotics in the treatment of tuberculosis and the reports of ototoxicity. The magnitude of the association was expressed as the reporting odds ratio (ROR), with 95% confidence intervals (CI). The ROR was used to determine relative risks for ototoxicity. RORs of ototoxicity and the suspected drug revealed that capreomycin administered to 117 patients showed an ROR for deafness of 1.4 (CI = 0.4–4.5) relative to streptomycin, which was the index drug for comparison. Amikacin and kanamycin were found to have much higher RORs than capreomycin (13). Overall, the reported incidence of hearing loss from capreomycin ranges from 0.7 to 25% (6).

A recent large scale meta-analysis of 35 studies comprising 9,178 patients with reports of significant adverse events associated with treatment of rifampin-resistant or MDR-TB was carried out using data from 53 centers in more than 30 countries. The highest rates of adverse effects leading to permanent drug discontinuation were found in patients treated with injectable antibiotics including aminoglycosides, capreomycin, amino-salicylic acid and linezolid. Of 1,932 patients treated with capreomycin, 161 experienced adverse events that resulted in stopping treatment. Only 71 cases reported specific adverse effects resulting in discontinuation of treatment. Twelve of 71 (17%) of those treated with capreomycin suffered hearing loss (14). Considering the large number of patients treated with capreomycin, the small number of persons experiencing hearing loss raised the question of statistical noise in the conclusion that capreomycin was responsible for the hearing loss reported.

### Animal Studies and Mechanisms of Ototoxicity

Early animal studies showed that long-term treatment with capreomycin in dogs and cats resulted in deafness in both species and vestibular injury in cats, demonstrated by reduction in post-rotatory nystagmus and severe ataxia just prior to death (15). The exact mechanism for capreomycin ototoxicity is unknown. There may be a common mechanism for capreomycin and aminoglycoside ototoxicity. Capreomycin has some features similar to those involved in aminoglycoside ototoxicity: induction of misreading in mitohybrid ribosomes and increased susceptibility of mutant mitohybrids with the A1555G and C1494U deafness alleles to drug action. But, these two classes of antibiotics differ in their activity on prokaryotic and eukaryotic ribosomes (2).

## Erythromycin Ototoxicity

### Clinical Studies

Erythromycin is an antibiotic that was isolated from a soil sample from the Philippines (16). It was isolated in 1952 from *Streptomyces erythreus* (17). This was the first drug in a new class of antibiotics called macrolides. Erythromycin is used to treat various infections including pneumonia caused by *Legionella pneumophila*. Prior to release for clinical use, it was tested for vestibular toxicity, but not for auditory toxicity, so it was presumed to be non-ototoxic. However, many cases of hearing loss have been reported since the first cases were reported in 1973 (18). They reported two patients who developed transient bilateral SNHL following intravenous administration of erythromycin lactobionate (18).

At least 50 to 100 cases of SNHL from erythromycin have been reported up to 2003 (19). Symptoms associated with ototoxicity include blowing tinnitus, vertigo and loss of hearing. Many of these patients were elderly or had liver or kidney disease. Hearing loss often begins within a few days after starting erythromycin. The audiometric pattern is usually bilaterally symmetrical, relatively flat 40- to 50-dB SNHL with good speech discrimination (19). However, some cases show a down-sloping pattern. Most patients with hearing loss experience resolution within 1–3 weeks after discontinuation of erythromycin (20). Other less common symptoms of erythromycin ototoxicity include vertigo and tinnitus. Some cases of SNHL were reported with the use of high dose intravenous erythromycin for Legionnaire's disease (21). A review of 20 cases reported in the literature revealed factors which, when combined with the use of high-dose erythromycin (≥2 g/day), might place patients at risk for erythromycin ototoxicity. These factors include: preexisting renal or hepatic disease, higher dosage and advanced age (22). In liver or kidney transplant patients, dose-related hearing loss has been reported. Patients receiving 2 g daily were found to have hearing loss in 16%, whereas 53% of patients treated with 4 g a day had hearing loss. These hearing losses were all reversible (23). Among liver transplant recipients, cyclosporine may have interacted with erythromycin to cause hearing loss (24). A patient who undergoing hemodialysis received 5 doses of 1 g of erythromycin lactobionate every 6 h then complained of hearing loss confirmed by audiogram. Serum concentrations of erythromycin were up to 100 mg/L and serum half-life was more than three times longer than normal. Hearing recovered after stopping the erythromycin (25). Hearing loss was reported in several other patients undergoing hemodialysis (26–30). Most cases of SNHL were reported in patients receiving intravenous erythromycin.

When erythromycin is combined with other ototoxic drugs, severe hearing loss may occur. Two cancer patients with liver dysfunction had severe bilateral SNHL following intravenous erythromycin lactobionate that gradually improved when the erythromycin dose was reduced. Both individuals were receiving other ototoxic drugs at the same time. One received gentamicin concomitantly and the other person was treated with tobramycin (31). A patient who was treated with intravenous erythromycin gluceptate in addition to gentamicin suffered a 55–60 dB SNHL in the speech range that later improved by about 20 dB (32). Exacerbation of hearing loss was reported in a patient who suffered high frequency SNHL from previous treatment with cisplatin. Hearing recovered to baseline levels after discontinuation of erythromycin (33).

Most reports of transient hearing loss from erythromycin occurred after intravenous injection. However, reversible SNHL has also been shown in a patient in severe renal failure who received high doses of oral erythromycin (30).

Permanent ototoxicity from erythromycin can occasionally occur. Two patients with persistent hearing loss after treatment with intravenous erythromycin lactobionate. One case complained of persistent tinnitus lasting at least 1 year and SNHL of 20–30 dB (34). The second patient was a 73 year old who had persistent hearing loss after receiving this drug (35). Permanent vestibular damage has been reported after intravenous erythromycin lactobionate in a patient with normal liver and kidney function despite having blood levels in the normal range. The patient also had SNHL that recovered after discontinuing erythromycin, but vestibular dysfunction persisted (36).

### Animal Studies and Mechanisms of Ototoxicity

The exact site and mechanism of erythromycin ototoxicity remain unknown (19). Auditory brainstem response testing was performed to determine site of lesion associated with erythromycin ototoxicity. Two cases of erythromycin-induced hearing loss were reported from whom, serial evoked auditory brainstem potentials (EABPs) were obtained. These records showed absence of waves I–III during treatment with erythromycin. All EABP waves became normal after erythromycin was discontinued (37).

Two studies of erythromycin ototoxicity in experimental animals were reported. These investigations suggested that erythromycin causes transient dysfunction of the stria vascularis. Intravenous erythromycin administered to guinea pigs resulted in a dose-dependent, transient partial decrease of the endocochlear potential (EP) and cochlear microphonics (CM) in the basal turn of the cochlea. Complete recovery of both EP and CM within 20 min after erythromycin dose was completed. In contrast to the effects of intravenous injection, the perilymphatic perfusion of erythromycin resulted in nearly complete loss of the CM and the decrease of both CM and EP was irreversible (38). These findings suggested that erythromycin caused hearing loss by interfering with ion transport mechanisms in the stria vascularis. Erythromycin caused a reversible decrease in the transepithelial short circuit current, a measure of the K+ secretion rate when perfused on the basolateral side of both stria vascularis marginal cells and homologous vestibular dark cells *in vitro* with a micro-Ussing chamber. These findings are consistent with the hypothesis that erythromycin ototoxicity is mediated, at least in part, by inhibition of K+ secretion in the inner ear (39). Some hearing losses associated with erythromycin were associated with central nervous system symptoms suggests that part of the hearing loss may be central (31).

The only published human temporal bone study of erythromycin ototoxicity presents findings that are consistent with the experimental findings in guinea pig cochlea discussed above. The finding of strial edema in all cochlear turns in the human temporal bone specimen suggests that strial dysfunction resulting from inhibition of K+ secretion could account for the observed hearing loss. Stria vascularis dysfunction could explain the flat threshold loss with good speech discrimination that is frequently found in patients with erythromycin ototoxicity, and it can also account for the reversible nature of the hearing loss (19).

Guidelines have been issued for prevention of erythromycin ototoxicity: (1) elderly individuals and patients with reduced liver or kidney function should have pretreatment audiograms and follow-up testing during or after treatment if symptoms occur; (2) care should be taken when erythromycin is treatment is combined with other ototoxic drugs; (3) doses of erythromycin should not exceed 1.5 g daily if serum creatinine is >180 mol/L (21).

The decline in the reported incidence of ototoxicity from erythromycin in recent years may result from adherence to the above guidelines and the development of new macrolide antibiotics like azithromycin and clarithromycin that have probably supplanted the use of erythromycin to a large extent.

## Azithromycin Ototoxicity

### Clinical Studies

Azithromycin and clarithromycin are alternatives to conventional macrolides like erythromycin in the routine treatment of many dermatologic, upper respiratory, and lower respiratory tract infections. They are tolerated better, less toxic, and more convenient to take. Their use may provide better patient compliance (40).

A clinical trial of 29 HIV negative patients, receiving only azithromycin 600 mg daily for 4 months followed by 2 months of azithromycin plus streptomycin for the treatment of MAC-associated lung disease. Three patients treated with azithromycin alone complained of hearing loss confirmed by audiograms. Hearing loss resolved after reducing the dose of azithromycin. Another 9 patients suffered hearing loss. However, they were also receiving streptomycin for 2 months (41). A group of 39 elderly, HIV- patients were treated with azithromycin for mycobacterial lung disease. Ten of 39 patients (26%) complained of hearing loss confirmed with audiograms. When the dose was reduced, hearing loss resolved. Patients with hearing loss had higher blood levels of azithromycin than those that did not (42). They found that the most common reason for dosage reduction or drug discontinuation with high-dose azithromycin was temporary hearing impairment (50%). Two patients developed reversible sudden SNHL during the use of low-dose azithromycin (43).

Many patients experienced reversible SNHL following prolonged high-dose therapy for *Mycobacterium avium* infections related to acquired immunodeficiency syndrome (AIDS). An HIV positive patient was treated with azithromycin 500 mg daily for several months for *Mycobacterium avium* complex (MAC) and *Mycobacterium kansasii*-associated pneumonia. He developed decreased hearing, bilateral tinnitus, and dizziness. An audiogram showed moderate to severe SNHL that improved over 3 weeks after stopping azithromycin (44). Among a group of 21 HIV+ patients treated with azithromycin 500 mg daily for MAC, 3 (14%) developed hearing loss that developed 4–12 weeks after initiation of azithromycin. The hearing loss was moderately severe in two patients and mild in the other. Hearing recovered 2–4 weeks after stopping the drug (45). A retrospective study of 46 HIV- patients with MAC infections treated with chronic azithromycin for up to 46 weeks revealed that 8 (17%) had ototoxic symptoms including hearing loss (88%), tinnitus (37%) or vertigo (25%). Audiograms revealed mild-to-moderate SNHL in four patients that were tested. These symptoms presented over a wide range of time (1.5–20 weeks) and resolved within 11 weeks after the drug was stopped (46).

A large randomized placebo controlled clinical trial was carried out with a large number of patients with chronic obstructive pulmonary disease (COPD). They were treated with azithromycin 250 mg daily for 1 year to test the hypothesis that this drug would decrease the frequency of acute exacerbations. SNHL was confirmed by audiogram in 142 (25%) of subjects receiving azithromycin, compared with 110 patients who received placebo (20%) (*p* = 0.04). Hearing improved to baseline in 21/61 (34%) patients who discontinued azithromycin (47).

Complete deafness was reported in a patient after treatment with intravenous azithromycin (48). A recent study comparing a short-term course of treatment in adults with uncomplicated infections with azithromycin vs. short-term use of amoxicillin ± clavulanate did not show an increased risk of SNHL (49). However, irreversible SNHL was reported in a patient treated with low-dose oral azithromycin prescribed for acute otitis media (50). Also a healthy 39-year-old woman complained of tinnitus and hearing loss after taking oral azithromycin for only 2 days for a urinary tract infection. Audiometry demonstrated a moderate-to-severe high frequency SNHL in the right ear and a mild-to-moderate high-frequency SNHL on the left. Speech discrimination was 92 and 96% on the right and left, respectively. Hearing loss persisted when the patient underwent a follow up audiogram after 1 year (51).

Patients with chronic lung diseases treated with long-term azithromycin were evaluated for adverse effects, including ototoxicity. MEDLINE and other databases were searched for relevant articles published up to August 2013. Randomized controlled trials were tabulated and azithromycin-related ototoxic events were included in the meta-analysis. Patients treated with long-term azithromycin therapy were found to have increased risk of hearing impairment. Their relative risk, (RR) was 1.168 [95% confidence interval (CI) = 1.030, 1.325]) (52). An analysis of adverse drug reports (ADR) from the Italian spontaneous reporting system (SRS) from 2001 to 2107 describing ototoxicity was carried out. The authors calculated the reporting odds ratio (ROR) using a case/non-case technique. Of a total of 325,980 reports 652 had at least one ototoxicity ADR. Azithromycin was found to have a significant adjusted ROR of 10.23 (confidence interval, 5.3–29.79) for onset of hearing loss (53).

Caution is advised in the treatment of SARS-CoV-2 with the combination of azithromycin with hydroxychloroquine, since both drugs have been shown to be ototoxic (54).

### Animal Studies and Mechanisms of Ototoxicity

The ototoxicity of azithromycin was tested in guinea pigs. Oral administration of azithromycin caused a reversible reduction in transient evoked otoacoustic emissions (TEOAEs). These findings suggest that azithromycin may exert transient ototoxic effects on outer hair cells. The use of TEOAE monitoring may be helpful in clinical studies of patients receiving prolonged or high doses of azithromycin (55).

Another study utilized topical application of various concentration of azithromycin solutions to the middle ear. Inner hair cells in the basal turn were severely damaged to a greater extent than were the outer hair cells in a concentration-dependent manner (56).

Very little is known about the mechanisms of ototoxicity of azithromycin. Animal studies discussed above suggest that azithromycin may act on outer hair cells to cause transient dysfunction with systemic treatment. Topical middle ear application resulted in severe preferential damage to inner hair cells. No mechanisms have been proposed for azithromycin ototoxicity.

## Clarithromycin Ototoxicity

### Clinical Studies

Clarithromycin (6-O-methyl-erythromycin A) was developed in Japan in 1980 to improve upon the profile of erythromycin. It is acid stable, causes fewer gastrointestinal side effects, and has a longer half-life than erythromycin. It can be administered twice a day instead of four times a day (57). This increases the likelihood of patient compliance with clarithromycin compared with erythromycin. Hearing loss has also been associated with clarithromycin. In phase II and III studies of 3,768 patients, two patients with acquired immunodeficiency syndrome (AIDS) developed a mild reversible partial SNHL after long-term high dose clarithromycin therapy for *Mycobacterium avium* complex (MAC) infections (58). A study of 13 patients with AIDS and *Toxoplasma* species-associated encephalitis treated with 2 g of clarithromycin and 75 mg of pyrimethamine per day for 6 weeks was reported. Two patients (15%) demonstrated a mild, persistent SNHL on audiometric testing during the 2nd week of treatment. The authors attributed the ototoxicity to the high dose of clarithromycin (59). One hundred seventy-three patients with AIDS and disseminated *Mycobacterium avium intracellulare* received clarithromycin treatment. Baseline audiograms were abnormal in 27/50 patients tested. Hearing worsened in 6 of these 27 patients (22%) (60).

Forty-five HIV negative patients with *M. avium*-associated lung disease received treatment with clarithromycin (500–2,000 mg per day) either alone or in combination with rifampin, an aminoglycoside (one patient), a quinolone, clofazimine, isoniazid, ethambutol, pyrazinamide, or minocycline. Twelve of these patients demonstrated baseline hearing impairment. Several had been previously treated with streptomycin. Four of the 12 (33%) patients developed additional hearing loss during treatment that was not severe enough to discontinue drug therapy (61).

A multicenter open trial to examine the antimicrobial activity and clinical efficacy of clarithromycin against disseminated *M. avium* in 77 patients with late-stage AIDS. Baseline audiometry was performed in 27 patients. Five patients had pretreatment hearing impairment that remained stable throughout drug treatment. Three additional patients developed hearing impairment during clarithromycin treatment (4%). The drug was discontinued in one patient whose hearing problem had been diagnosed clinically, and his hearing was restored. The drug regimens of the remaining seven patients with hearing loss were not changed since the impairment was not severe (62).

Twenty-two HIV negative patients with *Mycobacterium avium intracellulare* complex (MAIC) disease were treated with clarithromycin (0.75–2g/day), minocycline and clofazamine for 15 months. Three patients (3/22) developed ototoxicity requiring a reduction in the dose of clarithromycin by 50% after a short interruption of treatment. Audiograms were performed before treatment and after 1, 2, 3, and 6 months of treatment and when patients showed signs of hearing deterioration (63). No details were provided about the duration of treatment in relation to the onset of hearing loss.

A number of case reports documented hearing loss associated with clarithromycin showed that some were reversible, but some were not. A 76 year old male received high dose clarithromycin for atypical pulmonary tuberculosis. He developed progressive bilateral SNHL within 4 days. Subjective hearing improved when the dose was reduced and after completion of clarithromycin treatment hearing was restored to normal (64).

A 50-year-old male experienced tinnitus in his right ear while being treated with clarithromycin. Pure tone audiometry showed a 60 dB loss at 4 kHz on the right and a 40 dB loss at 4 kHz on the left. 2 days after discontinuing clarithromycin tinnitus resolved and hearing returned to baseline (65). A pregnant 28 year old female was treated with clarithromycin for an upper respiratory infection. The patient experienced a sudden unilateral SNHL of >90 dB. Clarithromycin was discontinued and the patient was treated with low dose steroids and hearing returned to presumed normal baseline of 20–30 dB (66). A 23-month-old child with cervical lymphadenitis caused by *Mycobacterium avium* complex was treated with clarithromycin and ethambutol. 15 weeks after starting clarithromycin, a threshold of 50 dB HL at 4 kHz was demonstrated on sound field audiometry. Distortion product OAEs were absent at 4 kHz, implicating abnormal outer hair cell function. Audiological testing was normal 4 weeks after clarithromycin was discontinued (67).

Buruli ulcer is a neglected tropical infectious disease caused by *Mycobacterium ulcerans* that damages the skin and subcutis. An open-label, randomized, controlled, phase three trial evaluated the efficacy of a fully oral treatment with once daily rifampicin and clarithromycin 15 mg/kg extended release for 8 weeks compared with standard of care, rifampicin and streptomycin, using a non-inferiority design. Otovestibular toxicity was more frequent in patients receiving combined rifampicin and streptomycin than in patients receiving combined rifampicin and clarithromycin. Only one patient (1%) in the group treated with clarithromycin suffered hearing loss >25 dB and no patient had vestibular toxicity (68).

An 81 year old woman developed sensorineural deafness in the right ear after receiving low dose oral clarithromycin for COPD worsened by infection. Even though the drug was stopped after 3 days, SNHL persisted. Despite cessation of this drug after only 3 days of treatment, the hearing loss was found to be irreversible (69).

SNHL was reported in 3 of 42 (7%) pediatric patients receiving prolonged clarithromycin therapy for the treatment of non-tuberculous mycobacterium (NTM) cervicofacial lymphadenitis. Hearing loss developed between 25 and 63 days after initiation of treatment. HL was unilateral in two children and persisted in one child after treatment was discontinued (57). Based on their review of the literature and the addition of their three cases, the authors estimated that the risk of clarithromycin ototoxicity was 9% (16/184) in healthy adults and 22% (6/27) among HIV-positive individuals (57).

A recent study analyzed ADR reports of ototoxicity from the Italian spontaneous reporting system (SRS) from 2001 to 2017. The authors measured disproportionality by calculating the reporting odds ratios (RORs) and 95% confidence intervals (CIs) with a case/non-case methodology. Clarithromycin (3.95, 1.86–8.40) demonstrated a statistically significant disproportionality for the onset of hearing loss (53). Thus, clarithromycin has a significant tendency to cause hearing loss. However, the authors did not stipulate whether the hearing loss was temporary or permanent. Nevertheless, it would be prudent to perform audiologic monitoring in patients receiving clarithromycin, particularly if they are receiving long-term treatment (57) even though hearing impairment may be less severe and occur less frequently with high-dose clarithromycin compared to high dose azithromycin (44, 53).

### Animal Studies and Mechanism of Ototoxicity

The mechanism of ototoxicity associated with clarithromycin is unknown. Experiments in the guinea pigs administered clarithromycin 75 mg/kg i.v. reversible reduction of transient evoked otoacoustic emissions (TEOAEs) (*p* < 0.05). The temporary diminution of TEOAE could likely be attributed to the transient dysfunction of outer hair cells (OHCs) (55).

## Macrolides

### Clinical Studies

Publications dealing with ototoxicity risk from macrolides searched large data bases and came to opposite conclusions. A retrospective nested case-control study of a health claims database, the LifeLink (IMS, Danbury, CT) was carried out. A random selection of patients aged 15–60 years old from 2006 to 2014 was performed. The authors found an association between SNHL and macrolide. However, a similar risk was found for case controls treated with non-ototoxic antibiotics. The authors concluded that there did not appear to be an increased risk of SNHL in patients treated with macrolide antibiotics (70). However, these authors did not include children under the age of 15 nor did they study patients >60 years of age. Macrolide ototoxicity has been observed in children (57, 71) and published case reports suggest that the elderly and patients with HIV disease may be at higher risk of ototoxic reactions to all the commonly prescribed macrolide antibiotics, including azithromycin, clarithromycin, and erythromycin (44).

A systematic review of the literature was accomplished. The authors reviewed 44 publications, describing patients of all ages. Three were prospective and 41 were retrospective studies. The authors summarized 120 cases of hearing loss associated with macrolide drug administration. Seventy-eight cases were SNHL confirmed audiometrically. Nine additional publications described 42 cases of subjective patient-reported hearing loss that was associated with oral or intravenous macrolide administration at standard and elevated doses. The authors concluded that SNHL may follow macrolide exposure, even at standard oral doses (72). It appears that the incidence of hearing loss from macrolides is quite low but sporadic cases can appear. They urged that future research is necessary to provide greater insights into the mechanisms, incidence and prevalence of macrolide ototoxicity (72).

A recent review of ototoxic drugs in Italy showed a significant association of ototoxicity with the individual macrolides azithromycin and clarithromycin (53). These authors stressed the importance of utilizing spontaneous reporting databases as a valid tool to detect drug-induced ototoxicity. They concluded that their data are consistent with results from clinical trials and post-marketing data for the ototoxic drugs they reported (53).

### Animal Studies and Mechanism of Ototoxicity

This has been covered s under erythromycin, azithromycin and clarithromycin.

## Vancomycin

### Clinical Studies

Vancomycin has been in clinical use as a potent anti-staphylococcal antibiotic for over 60 years. Vancomycin use has increased dramatically in the past three decades to keep up with the increase in methicillin-resistant *Staphylococcus aureus* (MRSA) infections (73). Most early reports of ototoxicity were from the use of early, relatively impure, formulations of vancomycin. Twenty-eight reports of vancomycin-associated ototoxicity had been published from 1958 to 1988. It was not specified whether hearing loss was permanent or temporary. Few patients had follow-up audiometry (74).

As of 1989, it appeared that in all cases of permanent SNHL attributed to vancomycin ototoxicity the patient received an aminoglycoside antibiotic just before, during, or shortly after vancomycin. In those cases, vancomycin may have interacted with the aminoglycoside to cause ototoxicity, or that the hearing loss was caused by an aminoglycoside antibiotic alone. Thus, it appeared that vancomycin may cause ototoxicity primarily when administered with other ototoxic agents, like aminoglycosides (17).

However, more recent reports suggest that vancomycin alone is ototoxic. A retrospective study of 89 adult patients who had baseline and follow-up audiograms after approximately 27 days of vancomycin therapy demonstrated a 12% risk of high frequency SNHL (11 patients). Regression tree modeling revealed that for patients <53 years old, no patients had detectable hearing loss. However, among patients >53 years old, the incidence was 19% (*P* < 0.008). Three of these patients were receiving ototoxic medications (one on gentamicin and two on furosemide), but this incidence was not significantly different than that for the group of patients without audiometric testing (73).

A recent cross-sectional study was carried out at an academic medical center from 2012 to 2019. Patients who received IV vancomycin for >14 days and had baseline and follow-up weekly audiometry were investigated. Among 92 patients tested with at least two audiograms while on vancomycin 7 (8%) exhibited SNHL Two patients had mild hearing loss. Two suffered mild to moderate loss and three showed moderate to severe SNHL. No variables were correlated with SNHL, including age >40, pretreatment SNHL, vancomycin doses >4 g/day or elevated serum levels of vancomycin (75). Serum concentrations of >40 mg/L have been previously associated with reversible SNHL, whereas concentrations >80 mg/L in patients with renal impairment may be associated with permanent SNHL (76). An analysis of adverse drug reports (ADR) from the Italian spontaneous reporting system (SRS) from 2001 to 2107 that included ototoxicity as discussed above for azithromycin and clarithromycin reported an odds ratio for vancomycin of 6.72 (confidence interval 2.14–21.11) indicating significant ototoxicity associated with vancomycin (53).

Most cases of ototoxicity associated with vancomycin have been in patients treated with IV vancomycin. However, two cases of ototoxicity after oral administration have been reported when it was used to treat *Clostridium difficile* colitis (77, 78).

Another case of vancomycin ototoxicity occurred when vancomycin was administered by intrathecal injection to treat *Corynebacterium Jeikeium* meningitis in a patient with acute lymphocytic leukemia. The patient received two doses of intrathecal vancomycin. Hearing decreased after the first dose and complete SNHL occurred after the second intrathecal dose. Hearing loss was irreversible (79). Intrathecal vancomycin may have entered the cochlea via the cochlear aqueduct, resulting in high concentrations of vancomycin in the cochlea leading to irreversible hearing loss.

## Vancomycin in Neonates

Vancomycin is a first-line agent in the treatment of serious Gram-positive infections in the neonatal population (80). The reported ototoxicity of vancomycin in neonates has been varied. Vancomycin has a longer half-life in premature infants. Infants weighing <1,000 g had significantly larger volumes of drug distribution and consequently longer drug half-lives than larger premature infants, regardless of post-conceptual or actual age (81). Nevertheless, some reports failed to show ototoxicity among vancomycin treated neonates. Six hundred twenty-five patients were analyzed; 45 neonates failed hearing screening. Vancomycin was administered in 130 patients. Exposure to vancomycin was not related to failure to pass Automated-ABR screening (82). Vancomycin administered to pregnant women did not appear to cause hearing loss in their infants who were tested after birth (83). A 5 year study of 2,347 Otoacoustic Emisssions (OAEs) of neonates treated with vancomycin was carried out in New Zealand. OAE failure occurred in 22% (9/41) of neonates treated with vancomycin compared to 7% in neonates not receiving vancomycin or aminoglycosides. Thus, treatment with vancomycin was associated with a statistically significant increased incidence of ototoxicity (84). Very low birth weight neonates appear to have an even higher risk for hearing loss from vancomycin. A large group (4,739) of very low birth weight infants from the German Neonatal Network who were treated with vancomycin were shown to have a dose-dependent risk for abnormal hearing test at discharge and abnormal pure tone testing at 5 years of age. Those infants in the upper quartile for vancomycin dose had the highest risk of hearing loss (85). Therefore, it appears that neonates, particularly those with very low birth weight, should undergo pretreatment auditory testing and follow up testing after treatment with vancomycin.

### Animal Studies and Mechanism of Ototoxicity

The mechanisms that underlie vancomycin ototoxicity are unknown. Experiments in guinea pigs demonstrated significant augmentation of gentamicin ototoxicity by vancomycin. The maximum output of the alternating current (AC) cochlear potential showed a small change after gentamicin 50 mg/kg alone. However, when the same dose of gentamicin was combined with vancomycin, a highly significant diminution of the AC cochlear potential occurred. Animals treated with 100 or 200 mg/kg had no significant loss of OHCs, nor did those guinea pigs receiving 50 mg/kg of gentamicin sulfate alone. However, combinations of 100 or 200 mg/kg of vancomycin with gentamicin had a dramatic increase in the loss of cochlear OHCs.

Vancomycin combined with gentamicin did not increase the serum or perilymph levels of gentamicin. The authors postulated that vancomycin may cause a selective accumulation of gentamicin in hair cells by increasing the permeability of these cells (86). This hypothesis has not been proven. Another experimental study of vancomycin ototoxicity was performed in guinea pigs using gentamicin and neomycin as positive controls. Hearing function was tested with ABRs before and after intraperitoneal administration of vancomycin (75, 150, 300 mg/kg for 11–17 days), gentamicin (60 mg/kg for 26 days), neomycin (100 mg/kg for 17 days) and sodium chloride solution. Hearing thresholds were significantly elevated in guinea pigs treated with each of the two aminoglycosides. However, vancomycin did not alter auditory thresholds in guinea pigs treated with 75 or 150 mg/kg, which were proposed as clinically relevant doses. The authors concluded that treatment with moderate doses of vancomyin comparable to clinical dosing did not cause a specific ototoxicity unlike the aminoglycosides (87). There have been no reports of transtympanic administration of vancomycin either to patients or to experimental animals. Additional research is needed to elucidate the exact mechanisms of vancomycin ototoxicity and the enhancement of aminoglycoside ototoxicity by vancomycin.

## Chloramphenicol Ototoxicity

### Clinical Reports

Chloramphenicol was isolated from soil bacteria, an actinomycete, from compost soil. It was later synthesized (88). Chloramphenicol is active against gram-positive and gram negative organisms and against rickettsia, mycoplasma, and chlamydia (89). It has been used clinically by injection and by topical administration for ear infections.

A case report described a patient who suffered gradually progressive sensorineural hearing loss after systemic treatment with chloramphenicol. It began in one ear, but when the course of treatment was repeated, the second ear became involved. Hearing loss progressed to profound bilateral SNHL in this patient (90).

Additional cases have been reported. Forty-nine cases of ototoxicity were reported from the University of Benin in Nigeria. Almost half of these patients received chloramphenicol (21/49 or 43%). Most of the chloramphenicol treatments were parenteral. Hearing loss was severe to profound, bilateral and permanent in 66% of these cases (91). A later report from the same institution examining 79 cases of ototoxicity found only a single case of ototoxicity attributed to chloramphenicol (92). It is not clear why there was such a decline in the incidence of chloramphenicol ototoxicity at that institution. Drug induced ototoxicity that occurred over a 3 year period was studied in 156 patients in Nigeria. Chloramphenicol ototoxicity was the most common ototoxic drug that was implicated. Twenty-five patients suffered ototoxicity from chloramphenicol. However, this study did not provide detailed information about these patients. It was not stated how many patients received chloramphenicol during this 3 year period (93).

### Animal Studies and Mechanism of Ototoxicity

Chloramphenicol ototoxicity has been studied in guinea pigs and in cats. In guinea pigs that had the drug applied to the round window membrane, permanent loss of cochlear microphonics of up to 30 dB was recorded (94). Chloramphenicol sodium succinate at concentrations of 5% or greater in Ringer's solution caused an irreversible loss of CM responses (95). Severe damage to the hair cells, supporting cells and stria vascularis of the basal turn of the guinea pig was observed (96). Chloramphenicol powder blown into the middle ear of guinea pigs resulted in profound loss of cochlea sensitivity within 24 h (97).

Cats treated with saturated chloramphenicol solution on gelatin sponge on the round window demonstrated a permanent reduction of CM responses. However, the threshold elevations were not as severe as in the guinea pig (98).

The mechanism of chloramphenicol ototoxicity is unknown.

## Conclusion

It is important to be aware that antibiotics other than aminoglycosides can cause hearing loss. These antibiotics can play a critical role in the treatment of life-threatening infections in patients with infections caused by bacteria that are resistant to standard therapy. Additional research is needed to determine the mechanisms by which these drugs can cause ototoxicity.

## Author Contributions

LR conceived the idea for the review presented in this article and wrote the main text. VR and DM helped with research and compilation of the manuscript. All authors contributed to the article and approved the submitted version.

## Conflict of Interest

The authors declare that the research was conducted in the absence of any commercial or financial relationships that could be construed as a potential conflict of interest.

## Abbreviations

SNHL: Sensorineural hearing loss
EABPs: Evoked auditory brainstem potentials
EP: Endocochlear potential
CM: Cochlear microphonics
HIV: Human immunodeficiency virus
MAC: *Mycobacterium aviae* complex
MAIC: *Mycobacterium avium intracellulare* complex
AIDS: Acquired immunodeficiency syndrome
COPD: Chronic obstructive pulmonary disease
ADR: Adverse drug reaction
ROR: Reporting odds ratio
SRS: Spontaneous Reporting System
TEOAEs: Transient evoked otoacoustic emissions
SARS-Cov-2: Severe acute respiratory syndrome coronavirus-2
CI: Confidence interval
NTM: Non-tuberculous mycobacterial
MRSA: Methicillin-resistant *Staphylococcus aureus*
ABR: Auditory Brainstem Response
AC: Alternating current
OHCs: Outer hair cells
MDR-TB: Multidrug-resistant-tuberculosis
XDR-TB: extensively drug resistant tuberculosis
ICSRs: individual case safety reports
UMC: Uppsala Monitoring Centre.
